# Blocked Autophagy by miR-101 Enhances Osteosarcoma Cell Chemosensitivity *In Vitro*

**DOI:** 10.1155/2014/794756

**Published:** 2014-06-09

**Authors:** Zhiqiang Chang, Lifeng Huo, Kun Li, Yiming Wu, Zhenming Hu

**Affiliations:** ^1^The Department of Orthopaedics, The First Affiliated Hospital of Chongqing Medical University, Yuzhong District, Chongqing 400016, China; ^2^The Department of Cervical Surgery, The Second Affiliated Hospital of Inner Mongolia Medical University, Hohhot 010030, China; ^3^The Second Affiliated Hospital of Inner Mongolia Medical University, Hohhot 010030, China; ^4^The Department of Anesthesiology, The PLA 264 Hospital, Taiyuan 030001, China

## Abstract

The adjuvant chemotherapy, such as cisplatin, doxorubicin, and methotrexate has significantly improved survival of osteosarcoma patients. However, the chemoresistance which arose with the chemotherapy blocks achieving favorable outcomes for some patients and finally led to relapse or metastatic disease. Studies have shown paradoxical functions of autophagy in tumor development, which has been demonstrated by microRNAs. In the present study, we determined the involvement of autophagy during the chemotherapy of osteosarcoma cell line, U-2 OS, and further determined the regulation of miR-101 on the autophagy in the U-2 OS cells. Results demonstrated that doxorubicin treatment of U-2 OS cells induced significantly high level of autophagy-characteristic acidic vesicular organelles (AVOs), and induced significant autophagy related protein expression in U-2 OS cells. While the miR-101 could significantly reduce the doxorubicin-induced AVOs and block the autophagy related protein expression in U-2 OS cells. Moreover, the autophagy blockage by miR-101 sensitized the U-2 OS cells to doxorubicin treatment. In summary, miR-101 blocks autophagy during the chemotherapy in osteosarcoma cells and enhances chemosensitivity *in vitro*.

## 1. Introduction

Osteosarcoma (OS) is the most common primary malignant bone tumor, which usually occurred in children and adolescents [1]. The adjuvant chemotherapy, such as cisplatin, doxorubicin, and methotrexate [2], is frequently applied in treatment of OS and has significantly improved survival of osteosarcoma patients [3]. However, there are problems with the chemotherapy, including severe side effects, and what is more, the chemoresistance which arose with the chemotherapy blocked achieving favorable outcomes for some patients and finally led to relapse or metastatic disease [3, 4]. Thus, to overcome the significant obstacle, the research into the mechanism of chemotherapy resistance in OS should be focused on.

Autophagy is derived from the meaning of “eating of self” and constitutes a basic cellular process of both physiological and pathological importance [5]. Autophagy is the major pathway involved in the degradation of proteins and organelles, cellular remodeling, and survival during nutrient starvation [6]. It has been established as a key regulator of cellular homeostasis, promoting the controlled degradation of cytoplasmic material both at steady state and during nutrient deprivation [7]. Studies have shown paradoxical functions of autophagy in tumor [8, 9]. On one hand, autophagy is tumor-promoting in the development of tumor; conspiring with inflammation, autophagy promotes tumor growth [10]. Autophagic LC3B can be used as a prognostic marker in patients with breast cancer, which highlights the importance of autophagy in the biologic behavior of chemoresistant cancer cells [11]. On the other hand, autophagy is associated with tumor cell death. Tumor-promoting and suppressive roles of autophagy have been found in the same mouse model of lung cancer [12]. Moreover, autophagy has been indicated as a cell death and tumor suppressor mechanism [13]. Recently, autophagy has been shown to play important role in OS. Targeting HMGB1-mediated autophagy has been confirmed as a novel therapeutic strategy for osteosarcoma [14]. Therefore, further uncovering the role of autophagy in OS can lead to finding a novel strategy for OS treatment.

MiRs (miRNAs) are small noncoding RNA, consisting of about 22 nucleotides (nt) in length, and play a broad range roles in biological processes, including gene expression [15], apoptosis [16], and autophagy [17], such as the potent inhibitor role of miRmiR-101 in autophagy [18]. And what is more, miRs have shown to be potential in cancer therapy [19, 20]. MiRmiR-30a sensitizes tumor cells to cisplatinum via suppressing Beclin-1 mediated autophagy [21]. miRmiR-23b regulates autophagy associated with radioresistance of pancreatic cancer [22]. Given the high importance of autophagy in the chemoresistance of tumors and the significant regulatory role of miRs in autophagy, the deep research into the role of miR-regulated autophagy in cancer cells during chemotherapy might shade light on the mechanism of chemoresistance.

In this study, we showed that autophagy and the overexpression of autophagy-related proteins were induced by chemotherapy. And miR-101 blocked chemotherapy-induced autophagy in osteosarcoma cells. Moreover, the blocked autophagy by miR-101 sensitized osteosarcoma cells to chemotherapy. These results provided new insight into the potential role of miR-101 against chemotherapy resistance during the treatment of osteosarcoma.

## 2. Results

### 2.1. Chemotherapy Induces Autophagy and Expression of Autophagy-Related Proteins

**Figure 1:**
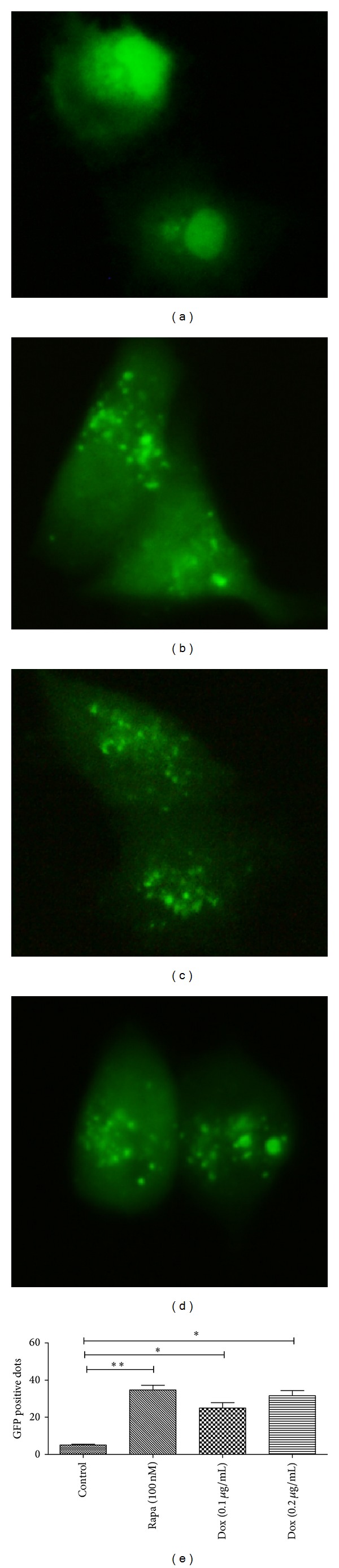
Dox promotes autophagic vesicles formation in U-2 OS cells. (a) Autophagic vesicles formation in negative control U-2 OS cells; (b) autophagic vesicles formation in U-2 OS cells which were treated with 100 nM rapamycin (positive control); (c) and (d) autophagic vesicles formation in Dox-treated U-2 OS cells (0.1 *μ*g/mL (c) or 0.2 *μ*g/mL (d)). Statistical significance shown as *P < 0.05, **P < 0.01. All experiments were independently performed at least for three times.

Autophagy is characterized by acidic vesicular organelles (AVOs) development [23]. The microphotographs of AVOs were observed via a fluorescence microscope and indicated by GFP-LC3 labeled autophagosomes. As an anticancer reagent, doxorubicin (Dox) has been widely used in clinical chemotherapy of various tumors, including OS. To deduce the chemoresistance of OS cells, we determined the autophagy in OS cells after Dox treatment. And it was shown in Figure 1 that the treatment of Dox with 0.1 or 0.2 *μ*g/mL induced significantly the high level of AVOs (GFP-positive dots) in one OS cell line, U-2 OS cells, compared to nontreated cells (cells treated with 100 nM Rapamycin (Rapa), one autophagy inducer as positive control).

**Figure 2:**
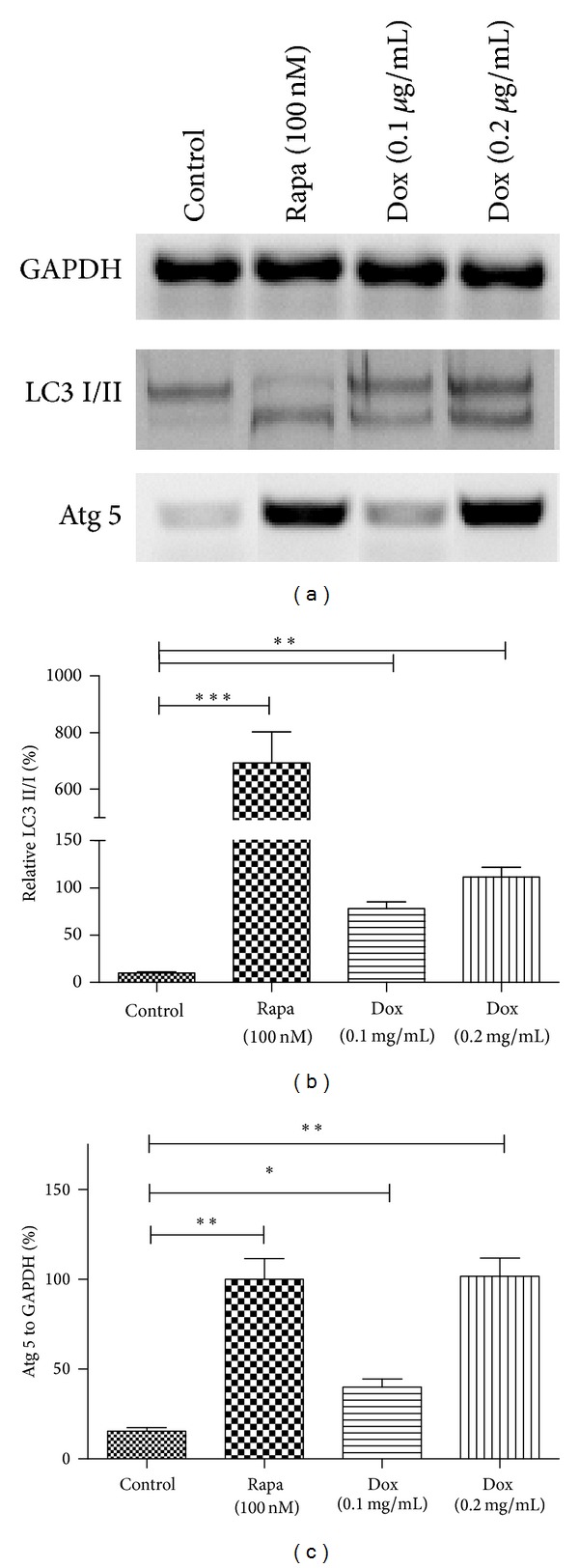
Dox promotes the conversion of LC3-I to LC3-II and increases Atg 5 expression. (a) Western blotting results of U-2 OS cells with Dox (0.1 or 0.2 *μ*g/mL) or Rapa (100 nM) treatment. (b) Quantitative analysis of the conversion of LC3-I to LC3-II in various groups. (c) Quantitative analysis of the relative Agt 5 expression to GAPDH in U-2 OS cells after various treatments. Statistical significance shown as *P < 0.05, **P < 0.01, and ***P < 0.001. All results were from three independently performed experiments.

Except for AVOs forming, autophagy is also characterized by the high conversion of LC-I to LC-II, both of which are two forms of LC3 protein and play important role in autophagosome formation [24]. To confirm the conversion of LC-I to LC-II, we used western blotting to analyze the lysates of US-2 OS cells with various treatments. As shown in Figures 2(a) and 2(b), there was a significant high level of LC3-II, compared to LC3-I, in U-2 OS cells treated with Rapa or Dox (0.1 *μ*M or 0.2 *μ*M). In addition, Figures 2(a) and 2(c) showed that the expression of the autophagy related protein 5 (Atg-5) increased significantly in Rapa or Dox treated U-2 OS cells. Taken together, these results indicated that Dox treatment induces autophagy in U-2 OS cells.

### 2.2. miR-101 Blocks Chemotherapy-Induced Autophagy in Osteosarcoma Cells

**Figure 3:**
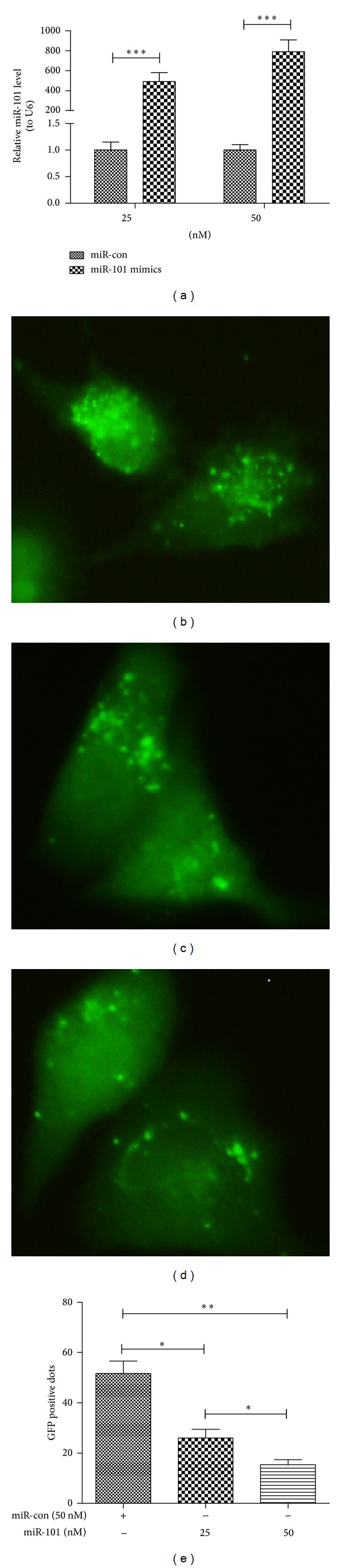
MiR-101 inhibits the Dox-promoted autophagic vesicles formation in U-2 OS cells. (a) MiR-101 mimics' transfection dramatically elevated the miR-101 level than the transfection of miRNA control. (b) AVOs formed in U-2 OS cells after 50 nM miR control transfection; (c) and (d) AVOs formed in U-2 OS cells after 25 (c) or 50 nM (d) miR-101 mimics transfection. (e) Quantitative analysis of GFP-positive dots (AVOs) in U-2 OS cells. (*P < 0.05, **P < 0.01). All experiments were independently performed at least for three times.

Chemotherapy increased autophagy and the expression of autophagy related proteins in osteosarcoma cell line, U-2 OS. And previous study indicated that miR-101 is a potent inhibitor to autophagy [18]. To establish a direct link between miR-101 and chemotherapy-induced autophagy, we examined the ability of miR-101 to regulate chemotherapy-induced autophagy. Firstly, we manipulated the miR-101 level in U-2 OS cells. As shown in Figure 3(a), the miR-101 level in U-2 OS cells was significantly elevated when cells were transfected with the miR-101 mimics (25 or 50 nM). Then we determined whether there was a regulation of miR-101 mimics on the Dox-induced autophagy in U-2 OS cells. As shown in Figures 3(b)–3(e), the miR-101 mimics transfection with 25 or 50 nM significantly reduced the AVOs formation more than the miR control transfection in U-2 OS cells. And what is more, the GFP positive dot number reduction was associated with the miR-101 mimics concentration (Figures 3(c)–3(e)).

**Figure 4:**
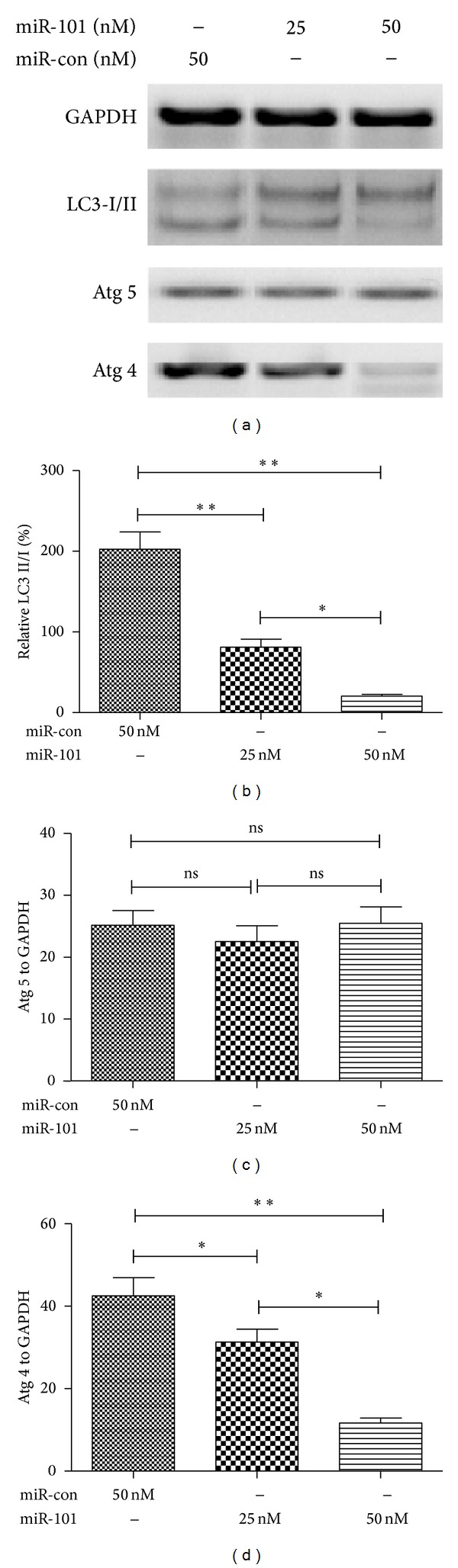
MiR-101 inhibits conversion of LC3-I to LC3-II and inhibits Atg 4 expression. (a) Western blotting results of Dox-treated U-2 OS cells simultaneously with miR control or miR-101 mimics transfection. (b) The conversion of LC3-I to LC3-II after miR-101 mimics or miR control transfection; (c) and (d) the percentage of Atg 5 or At 4 expression to GAPDH after miR-101 or miR control transfection (*P < 0.05, **P < 0.01, and ***P < 0.001). All results were from three independently performed experiments.

We also examined the effects of miR-101 mimics transfection on the expression of autophagy-related proteins in U-2 OS cells. The western bloting assay was also used in analyzing the proteins associated with miR-101 transfected U-2 OS cells. The Doc-induced conversion of LC3-I to LC3-II was inhibited by the transfection of miR-101 mimics (Figures 4(a) and 4(b)). However, the expression of Atg 5 had no significant differences between miR control and miR-101 mimics (25 or 50 nM) transfection groups (Figures 4(a) and 4(c)). The expression of another autophagy related protein, Atg 4, was decreased in the miR-101 transfected U-2 OS cells, compared to the miR-con transfected cells (Figures 4(a) and 4(d)). These results show miR-101 blocked chemotherapy-induced autophagy and expression of autophagy-related proteins in OS cells.

### 2.3. Blockage of Autophagy by miR-101 Sensitizes Osteosarcoma Cells to Chemotherapy

**Figure 5:**
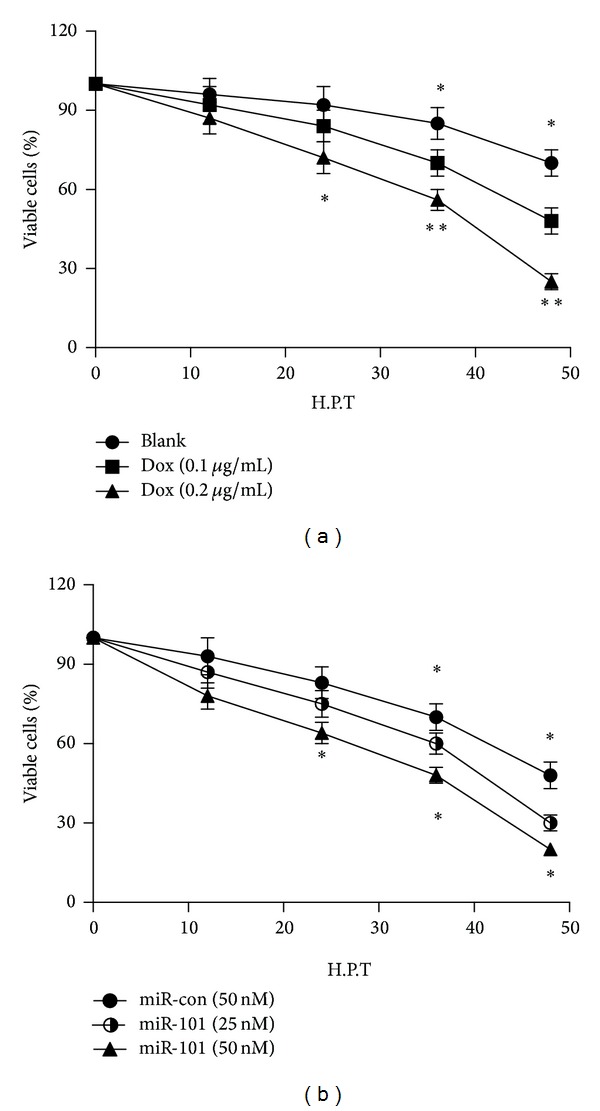
MiR-101 deteriorates the Dox-induced cell viability reduction. (a) Viability of U-2 cells after Dox treatment with 0, 0.1, or 0.2 *μ*g/mL. (b) Viability of U-2 cells after 0.1 *μ*g/mL Dox treatment and the transfection with 25 or 50 nM miR-101 mimics or 50 nM miR control (*P < 0.05; **P < 0.01). All results were from three independently performed experiments.

To further determine the influence of the inhibition of autophagy by miR-101 on the prognosis of U-2 OS cells, we determine the viability of U-2 OS cells after Dox treatment alone or Dox treatment along with miR-101 mimics transfection, by performing MTT assay.  Figure 5(a) demonstrated that Dox treatment significantly reduced the viability of U-2 OS cells, compared to the untreated cells. And the treatment with 0.2 *μ*g/mL caused a more severe cell viability reduction than the treatment with 0.1 *μ*g/mL, showing a dose-dependent effect. Moreover, the MTT assay demonstrated that the miR-101 mimics transfection deteriorated the Dox-induced (0.1 *μ*g/mL) viability decrease in U-2 OS cells (Figure 5(b)). A dose-dependence was also demonstrated in the cell viability deterioration caused by miR-101 mimics transfection (Figure 5(b)). These results reveal that blocked autophagy by miR-101 deteriorates the Dox-induced U-2 OS cell viability reduction.

## 3. Discussion

Remarkable cure rate has been elevated by chemotherapy in various human malignancies including osteosarcoma. However, chemoresistance has been shown to be one of the main obstacles to the elevation with various mechanisms [25]. Recently, researchers have focused on autophagy regulation of many cell processes related to tumors. Autophagy plays an important role in cellular homeostasis regulation, including degradation of proteins and organelles, preventing the toxic accumulation of damaged components and cellular remodeling. Overcoming chemoresistance remains a key challenge in chemotherapy of cancer.

In this study, we chose the U-2 cell line of OS to determine whether autophagy was induced by chemotherapy and the role of blocked autophagy by miR-101 in the chemotherapy of osteosarcoma. We investigated that chemotherapy induces autophagy characterized via AVOs which was revealed by GFP-LC3 with a microscopy microphotography. Comparing with the control group, the dox treated groups shows high level of AVOs (Figure 1); significantly high levels of LC-3 and Atg 5 were expressed in the Rapa or Dox groups (Figure 2). These findings demonstrated that autophagy could be induced by chemotherapy in U-2 OS cells* in vitro*.

Researches show that miRs are key regulators of many cell processes; miRs are a large group of non-coding RNA which regulate gene expression and biological processes including apoptosis [16] and autophagy [17]. The research of miR-101 regulate autophagy in OS cells has rarely been examined. We measured chemotherapy-induced autophagy in OS cells which were inhibited by the transfection of miR-101. The miR-101 not only decreases the formation of autophagic vesicles (Figure 3) but also reduces the expression of LC-3II and Atg 4. This part of the study shows that miR-101 blocks chemotherapy-induced autophagy in OS cells.

The viability of U-2 cells pretreated by Dox and after being transfected by miR-101 was demonstrated by MTT assay.  Figure 5 shows that cell surviving was significantly deteriorated after transfected by miR-101. The MTT assay also figures out that the sensitivity of OS cells to chemotherapy is increased by miR-101 blocked autophagy.

In summary, our study confirms that chemotherapy can induce autophagy; miR-101 blocked the chemotherapy induced autophagy, and the blocked autophagy by miR-101 enhances the sensitivity of the OS cell line U-2* in vitro*.

## 4. Materials and Methods

### 4.1. Cell Culture and Reagents

Human osteosarcoma cell lines (U-2 OS) were obtained from the cell resource center of the Chinese Academy of Medical Sciences. The cells were grown in RPMI-1640 (Invitrogen, Carlsbad, CA, USA) medium supplemented with 10% fetal calf serum (FCS) (GIBCO, Rockville, MD, USA), 50 *μ*g/mL streptomycin (Sigma-Aldrich, St. Louis, MO, USA). Cells were incubated in a humidified atmosphere at 37°C in 5% CO_2_. The antibodies to GAPDH, LC3, Atg 5, or Atg 4 were purchased from Santa Cruz Biotechnology (Santa Cruz, CA, USA). Rapamycin (Rapa) and doxorubicin (Dox) were purchased from Sigma-Aldrich (St Louis, Mo, USA). Secondary goat anti-rabbit IgG (Pierce, Rockford, IL, USA) was used to detected the specific binding of antibodies to their antigens.

### 4.2. Quantitative GFP-LC3 Analysis

Quantitative GFP-LC3 analysis in U-2 OS cells was conducted by transfecting with a GFP-LC3-expressing plasmid using Lipofectamine 2000 (Invitrogen, Carlsbad, CA, USA). After transfection for 24 h, cells were cultured in RPMI-1640 medium containing 1% FBS, rapamycin, or doxorubicin for another 24 h. In the following experiments, cells were additionally transfected with miR-101 mimics or miR control simultaneously. Images of GFP-positive dots were analyzed by a fluorescence microscopy.

### 4.3. Western Blot Analysis

Cells were washed with PBS and cell extracts were prepared by a standard protocol. Protein expression was determined by western blotting as previously described [26]. Protein samples were firstly separated by a 8–12% SDS-PAGE and transferred to a nitrocellulose membrane. And then the membrane was inoculated with primary rabbit polyclonal antibodies against LC3, GAPDH, Atg 4, or Atg 5 and followed by the secondary goat anti-rabbit IgG (Pierce, Rockford, IL, USA) inoculation. Finally, the results were analyzed by an enhanced chemiluminescence detection system (SuperSignal West Femto; Pierce).

### 4.4. *In Vitro* Viability Assay

Cell viability was determined by MTT assay. Cells were seeded in 96-well plates and attached overnight. Afterwards, cells were treated with or without 0.1 or 0.2 *μ*g/mL Dox, and after 12, 24, 36, or 48 h inoculation, cell viability was measured. The MTT assay was conducted according to the standard protocol. Absorbance was measured at 570 nm with a reference wavelength of 750 nm using a spectrophotometer. In another experiment, cells were treated with 0.1 *μ*g/mL Dox and were transfected with 25 or 50 nM miR-101 or 50 nM miR control using Lipofectamine 2000. And after 12, 24, 36, or 48 h of inoculation, cell viability was measured.

### 4.5. Statistical Evaluations

All statistical evaluations are presented as mean ± SE. The data were analyzed by Student's *t*-test, and the criterion for statistical significance was considered as *P* < 0.05 or less.
